# Cas9-Assisted Targeting of CHromosome segments CATCH enables one-step targeted cloning of large gene clusters

**DOI:** 10.1038/ncomms9101

**Published:** 2015-09-01

**Authors:** Wenjun Jiang, Xuejin Zhao, Tslil Gabrieli, Chunbo Lou, Yuval Ebenstein, Ting F. Zhu

**Affiliations:** 1 School of Life Sciences, Center for Synthetic and Systems Biology, MOE Key Laboratory of Bioinformatics, Collaborative Innovation Center for Diagnosis and Treatment of Infectious Diseases, Tsinghua University, Beijing 100084, China; 2 CAS Key Laboratory of Microbial Physiological and Metabolic Engineering, Institute of Microbiology, Chinese Academy of Sciences, Beijing 100101, China; 3 Raymond and Beverly Sackler Faculty of Exact Sciences, School of Chemistry, Tel Aviv University, Tel Aviv 6997801, Israel

## Abstract

The cloning of long DNA segments, especially those containing large gene clusters, is of particular importance to synthetic and chemical biology efforts for engineering organisms. While cloning has been a defining tool in molecular biology, the cloning of long genome segments has been challenging. Here we describe a technique that allows the targeted cloning of near-arbitrary, long bacterial genomic sequences of up to 100 kb to be accomplished in a single step. The target genome segment is excised from bacterial chromosomes *in vitro* by the RNA-guided Cas9 nuclease at two designated loci, and ligated to the cloning vector by Gibson assembly. This technique can be an effective molecular tool for the targeted cloning of large gene clusters that are often expensive to synthesize by gene synthesis or difficult to obtain directly by traditional PCR and restriction-enzyme-based methods.

## Abstract (web summary)

Genomic engineering often requires the cloning of long DNA segments that contain large gene clusters. Here, the authors describe an RNA-guided Cas9 nuclease assisted *in vitro* technique that allows the targeted cloning of near-arbitrary, long bacterial genomic sequences of up to 100 kb in a single step.

## Graphical abstract

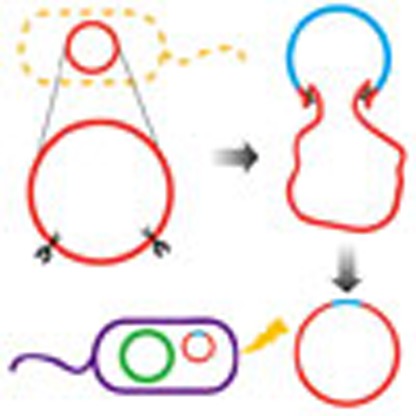

Cloning long genome segments of large gene clusters is an imperative step in engineering organisms for the purposes of producing high-added-value biomolecules such as pharmaceuticals and biofuels^1^,^2^,^3^,^4^,^5^,^6^. Traditional PCR-based cloning methods are often limited by the sequence length and GC content of the DNA template: standard PCR reactions routinely yield fragments of up to 10 kb, while longer PCR products require tedious optimization of reaction conditions and, even under ideal conditions for special cases, are typically limited to 35 kb (ref. 7). Alternatively, one may generate long genomic sequences of interest through the assembly of multiple short fragments, such as overlapping PCR products or chemically synthesized DNA oligos, although such methods tend to be time-consuming and expensive, particularly for obtaining sequences longer than 50 kb (which typically require three to five stages, each containing multiple assembly events)^8^,^9^. Another route to obtain long genomic sequences is by restriction enzyme digestion of genomic DNA. However, being a non-targeted approach, selecting a specific sequence of interest from a vast number of restriction digest products can be intensely challenging and cumbersome^10^. Certain techniques such as transformation-associated recombination (TAR)^11^,^12^ and single-strand overlapping annealing^13^ have been developed to clone-specific, large bacterial gene clusters following restriction enzyme digestion. Nevertheless, the utility of these techniques remains limited because they rely on the availability of unique restriction sites that flank the target genomic region and often the presence of selection markers in the target sequence. To facilitate advancements in biotechnology and synthetic biology, it is crucial to develop a general approach to clone near-arbitrary, long genomic sequences that are difficult to obtain using conventional methods.

The CRISPR-Cas9 endonuclease can be directed by guide RNAs to cleave specific sequences of DNA targets^14^,^15^,^16^,^17^. In the case of the *Streptococcus pyogenes* Cas9 (spCas9), the guide RNA system consists of a CRISPR RNA (crRNA) and a *trans*-activating crRNA (tracrRNA), which can be fused to form a single guide RNA (sgRNA)^18^. The target sequence is composed of a crRNA-complementary protospacer sequence and a protospacer adjacent motif (PAM)^18^. Here the use of long (∼20 bp) and programmable protospacer sequences as recognition sites offers the spCas9 endonuclease system higher targeting specificity and versatility than traditional restriction enzymes (with fixed recognition sites limited to 4–8 bp). This has motivated extensive development of Cas9-based tools for genome-editing, sequence-specific control of gene expression, as well as imaging *in vivo*^19^,^20^,^21^. In contrast, the potential applications of the Cas9 system *in vitro* have not yet been well explored; instead, they mainly focused on testing the enzyme's cleavage efficiency and sequence-recognition specificity^22^,^23^.

Here we show that in addition to being a versatile genome-editing tool *in vivo*, the *in vitro* application of spCas9 brings unparalleled efficiency and simplicity to the cloning of large gene clusters, allowing the targeted cloning of near-arbitrary, long bacterial genomic sequences of up to 100 kb to be accomplished in a single step. The technique described below can be an effective molecular tool for the targeted cloning of large gene clusters that are often expensive to synthesize by gene synthesis or difficult to obtain directly by traditional PCR and restriction-enzyme-based methods.

## Results

### Design of the CATCH cloning method

**Figure 1:**
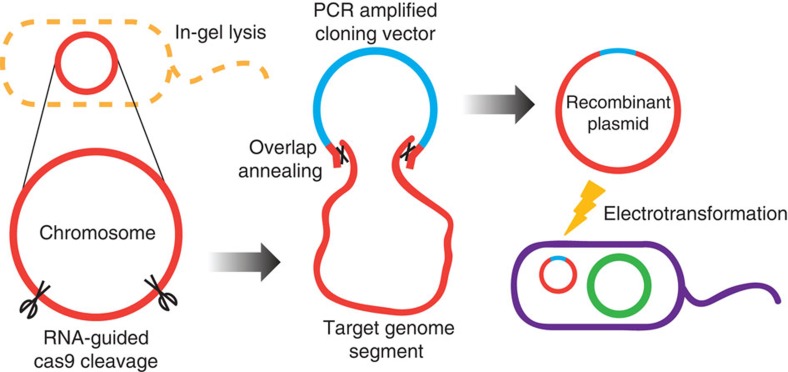
One-step large-gene-cluster cloning by CATCH. After in-gel lysis of bacterial cells, the chromosomes are cleaved by RNA-guided Cas9 at the designated target sites. A cloning vector (length not to scale) that shares 30-bp terminal sequence overlaps (black cross) with the target DNA at both ends is ligated to the target segment in a Gibson assembly mix. The recombinant plasmid is then electrotransformed into a cloning host.

In our cloning method by Cas9-Assisted Targeting of CHromosome segments (CATCH; Fig. 1), bacterial chromosomes after cell lysis are digested by RNA-guided Cas9 at designated target sites in agarose gel. The cloning vectors are designed so that they share terminal sequence overlaps (30 bp) with the target DNA at both ends, and are ligated to the target DNA through sequence complementarity in a Gibson assembly mix^9^. The recombinant plasmids are then electrotransformed into a cloning host. The procedure takes ∼8 h of bench time over 1–2 days to accomplish using standard laboratory equipment at reasonable costs, which drastically simplifies and accelerates efforts to clone large bacterial genomic sequences.

### Cleavage and isolation of long genome segments by CATCH

**Figure 2:**
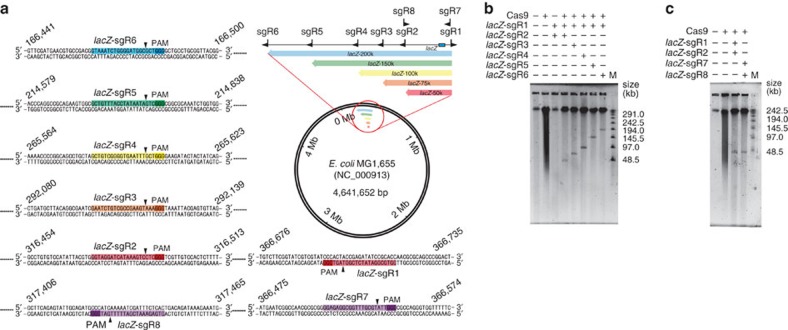
CATCH cleavage and cloning of long genomic sequences of various lengths. (**a**) A total of six sgRNA pairs were designed to target genome segments of different lengths (50, 75, 100, 150 and 200 kb, respectively, including one pair targeting 50 kb but with the PAM sequences falling on the flanking sequences instead of the target genome segment) in the *E. coli* str. K-12 substr. MG1655 genome, all containing a *lacZ* gene. The genome location, target sequences, PAM sequences and cleavage sites are detailed and highlighted. (**b**) *E. coli* chromosomes in agarose gel plugs were digested by Cas9 with the corresponding sgRNA pairs and analysed using PFGE. (**c**) A 50-kb segment on the *E. coli* chromosomes was targeted by two sgRNA pairs of opposite orientations, and the cleavage products were analysed using PFGE. M, marker.

To test the nuclease activity of Cas9 in agarose gel and its effectiveness in isolating long DNA sequences, we designed five sgRNA pairs to target segments of different lengths (50, 75, 100, 150 and 200 kb guided by *lacZ*-sgRNAs 1–6, respectively; see Methods) in the *Escherichia coli* genome, all containing a *lacZ* gene (Fig. 2a). After being embedded in low-melting-temperature agarose gel plugs, bacterial cells were treated by lysozyme and proteinase K, and washed by buffer successively to remove cellular components, leaving behind the genomic DNA. The intact chromosomes were protected by the agarose matrix, allowing for further manipulations with minimal mechanical shearing. The plugs were soaked in a reaction buffer containing pre-assembled Cas9 with the corresponding sgRNA pair and incubated at 37 °C for 2 h to allow for sufficient enzyme diffusion and digestion of genomic DNA in agarose gel. After digestion, 1/3 of a plug was cut out for pulsed-field gel electrophoresis (PFGE) to assess the cleavage efficiency (Fig. 2b). A clear band at the expected length was observed in each of the five lanes, while the control lanes showed either no band or heavy smear, suggesting sufficient cleavage specificity of the RNA-guided Cas9 in agarose gel.

To optimize the RNA-guided Cas9 in-gel digestion and to understand factors affecting the cleavage efficiency, we prepared agarose gel plugs of different volumes, each containing bacterial cells of different concentrations, and treated with different concentrations of Cas9 and sgRNA at different incubation time durations (Supplementary Fig. 1). We showed that bacterial cell concentration at 5 × 10^8 ^ml^−1^ was optimal for both cleavage and visualization on PFGE gel. We also showed that final concentrations of 0.02–0.1 mg ml^−1^ Cas9 was optimal for in-gel digestion (note that the enzyme activity may vary depending on the expression and purification methods used to obtain the enzyme). Notably, using a final concentration of sgRNA >30 ng μl^−1^ appeared to be essential for the in-gel digestion and Cas9 cleavage, and thus careful preparation of sufficient sgRNA is critical to the success of CATCH cloning. In addition, an incubation time of 1–2 h was sufficient for Cas9 diffusion and cleavage in gel plugs.

**Figure 3:**
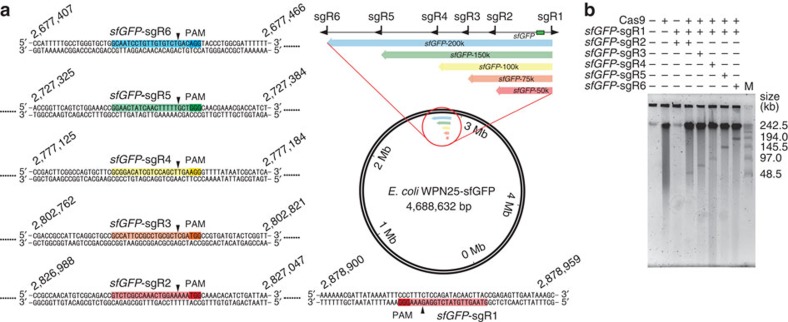
CATCH cleavage and cloning of long genomic sequences of various lengths from different genomic locations. (**a**) A total of five sgRNA pairs were designed to target segments of different lengths (50, 75, 100, 150 and 200 kb, respectively) in the *E. coli DH5a* str. WPN25-sfGFP genome, all containing an *sfGFP* gene. The genome location, target sequences, PAM sequences and cleavage sites are detailed and highlighted. (**b**) *E. coli* chromosomes in agarose gel plugs were digested by Cas9 with the corresponding sgRNA pairs and analysed by PFGE. M, marker.

**Figure 4:**
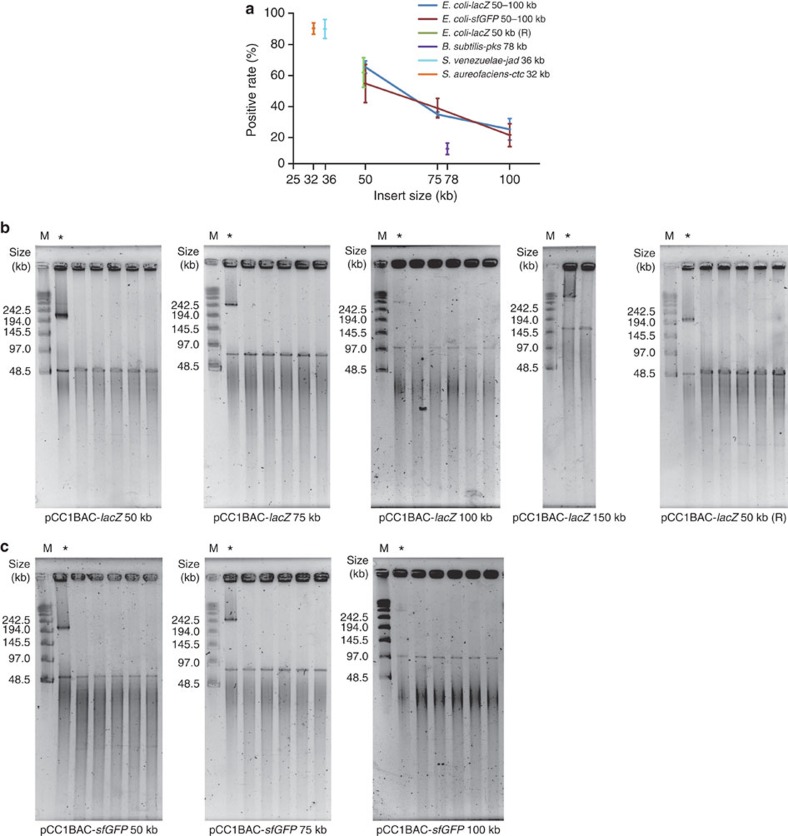
Efficiency of CATCH cloning of different genomic sequences. (**a**) The positive rates of CATCH cloning with different insert sizes and target sequences from different genome locations, as well as from different bacteria are analysed (error bar: s.d.; *n*=3). *E. coli-lacZ* 50 kb (R) is the cloning result of a 50-kb genome segment targeted by *lacZ*-sgRNAs 7 and 8, with the PAM sequences falling on the flanking sequences instead of the target genome segment. (**b**) Plasmids carrying the target sequences of different lengths cloned from *E. coli* str. K-12 substr. MG1655 (ligated to an 8-kb BAC vector) were purified from the blue–white-screening- and PCR-positive clones, linearized and analysed by PFGE. (**c**) Plasmids carrying the target sequences of different lengths cloned from *E. coli DH5a* str. WPN25-sfGFP were purified, linearized and analysed by PFGE. M, marker; asterisk, λ-terminase-free control.

Furthermore, we tested whether the orientation of sgRNA targeting could influence the cleavage and cloning efficiency, potentially affected by the post-cleavage exonucleolytic processing activity of Cas9 (ref. 18). We designed the *lacZ*-sgRNAs 7 and 8 to target the same 50-kb genome segment containing a *lacZ* gene as *lacZ*-sgRNAs 1 and 2 do, but with the PAM sequences falling on the flanking sequences instead of the target genome segment, leaving the two exonucleolysed ends on the target sequence (Fig. 2a). The cleavage and cloning experiments suggested that no apparent difference of cleavage efficiency (Fig. 2c) and cloning efficiency as a result of different sgRNA-targeting orientation was observed (Fig. 4a ‘*E. coli-lacZ* 50 kb (R)', Table 1; see results below). To test whether the location of target genome segments could affect the cleavage and cloning efficiency, we designed another five pairs of sgRNAs (*sfGFP*-sgRNAs 1–6) targeting five genome segments (50, 75, 100, 150 and 200 kb in length, respectively) at different genome locations (2,677,407–2,828,959 in the *sfGFP*-knock-in *E. coli* strain *E. coli DH5a* str. WPN25-sfGFP (Fig. 3a) versus 166,441–366,735 in the *lacZ*-gene-containing strain *E. coli* str. K-12 substr. MG1655 (Fig. 2a)). The results suggested that the cleavage efficiency (Figs 2b and 3b) and cloning efficiency (see results below) were unaffected when different locations of the genome were targeted.

### Cloning various long genome segments

Having successfully cleaved the long genomic sequences of interest from bacterial chromosomes, we attempted to ligate the target DNA into BAC vectors in a Gibson assembly mix^9^, taking advantage of the high sequence specificity of Gibson assembly, without the need for size selection on PFGE and gel purification from the background genomic DNA. We first prepared BAC vectors that shared 30-bp terminal sequence overlaps with the target DNA (Fig. 1; see Methods). The remaining of the Cas9-digested plugs (one- and two-thirds of gel plugs, after 1/3 of a plug has been used for PFGE from a total of two starting plugs) were pooled, melted and digested using agarase, after which the DNA was purified using ethanol precipitation and resuspension in nuclease-free water (see Methods). We then mixed the recovered DNA and vector in a Gibson assembly mix containing T5 5′–3′ exonuclease, *Taq* DNA ligase and high-fidelity polymerase. Finally, the ligation mix was electrotransformed into electrocompetent *E. coli* cells. Depending on the length of the target DNA to be cloned, we obtained 50–100 colonies on selective LB plates containing chloramphenicol, isopropyl-b-D-thiogalactoside (IPTG) and X-gal, among which 20–65% appeared blue (for detailed data, see Fig. 4a, Table 1 and Supplementary Table 2). All blue clones were selected and validated using PCR and sequencing at one of the two junction sites opposite to the *lacZ* gene (see Methods, Supplementary Fig. 2). The cloned BAC plasmids were purified, linearized and analysed with PFGE (Fig. 4b). All of the blue colonies appeared to be positive clones with correct insert sizes ranging from 50 to 150 kb; however, only one positive clone was obtained with the 150-kb insert and none with 200 kb (Table 1 and Supplementary Table 2).

We next tested whether the orientation of sgRNA targeting and the location of target genome segments could affect the cloning efficiency. We showed that the cloning efficiency was unaffected by different sgRNA-targeting orientations (Fig. 4a ‘*E. coli-lacZ* 50 kb (R)', Table 1 and Supplementary Table 2). We attribute these results in part to the robustness of the Gibson assembly that ensures the ligation with the target sequence. Furthermore, the cloning efficiency of genome segments of the same lengths but at different genome locations (166,441–366,735 in *E. coli* str. K-12 substr. MG1655 (Fig. 2a) versus 2,677,407–2,828,959 in *E. coli DH5a* str. WPN25-sfGFP (Fig. 3a)) were comparable (Fig. 4a, Table 1, Supplementary Fig. 2 and Supplementary Table 2). Taken together, our results suggested that the positive rates were size-dependent (where the cloning of DNA segments of longer than 100 kb was less efficient), but not genome-location- or sgRNA-orientation-dependent.

### Cloning large gene clusters from different bacteria

**Figure 5:**
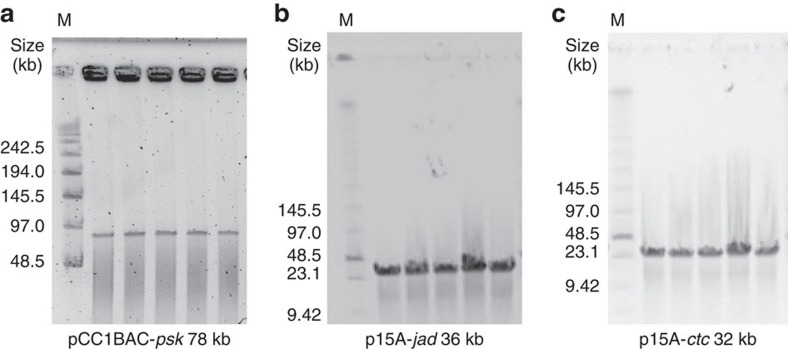
Cloning large gene clusters from different bacteria into different vectors. Plasmids carrying the target large gene clusters cloned from *B. subtilis* (**a**), *S. venezuelae* (**b**) or *S. aureofaciens* (**c**), respectively (see Table 1 and Supplementary Table 2), were purified from the PCR-positive clones, linearized and analysed using PFGE. M, marker.

Encouraged by the successful targeted isolation and cloning of genomic sequences from *E. coli* at various lengths, we turned to the cloning of large gene clusters from other bacterial genomes. Here we tested our method on cloning the 78-kb bacillaene-producing *psk* gene cluster (the largest gene cluster in *Bacillus subtilis*)^24^, a gene cluster of particular interest to chemical biologists for producing polyketides^5^,^6^, into BAC vector. Using the same method mentioned above, we obtained a total of 12 positive colonies in three trials with an ∼12% positive rate (Fig. 4a ‘*B. subtilis-pks* 78 kb', Fig. 5a, Table 1, Supplementary Fig. 2 and Supplementary Table 2). In addition, we cloned the 36-kb jadomycin-producing *jad* gene cluster from *Streptomyces venezuelae*^25^ and the 32-kb chlortetracycline-producing *ctc* gene cluster from *S. aureofaciens*^26^ into the p15A vector (Fig. 4a ‘*S. venezuelae-jad* 36 kb' and ‘*S. aureofaciens-ctc* 32 kb', Fig. 5b,c). Overall, we obtained ∼60 positive colonies in each experiment, with positive rates at ∼90% (Fig. 4a, Table 1, Supplementary Fig. 2 and Supplementary Table 2), demonstrating the versatility of CATCH on cloning various bacterial genomic sequences into different cloning vectors.

## Discussion

A long-standing challenge in traditional cloning is how to isolate and clone specific genomic sequences that are difficult to obtain using PCR or restriction-enzyme-based methods. Taking advantage of the RNA-guided CRISPR-Cas9 system *in vitro* to cleave and clone long DNA sequences, CATCH can synergize with gene synthesis, synthetic biology and chemical biology efforts for purposes such as producing biofuels, pharmaceuticals or other high-added-value biomolecules^1^,^2^,^3^,^4^,^5^,^6^.

So far, we have been able to efficiently clone bacterial genomic sequences of up to 100 kb, a sufficient length for most known bacterial gene clusters. We showed that CATCH cloning of DNA segments longer than 100 kb was less efficient, despite that each step (Cas9 digestion and DNA segment separation, Gibson assembly and BAC maintenance) alone could handle longer DNA fragments, suggesting that ∼150 kb could be a size limit to the CATCH cloning method itself with the current experimental set-up. The reduced cloning efficiency with longer inserts may in part result from mechanical shearing of long DNA fragments during manipulation, the lower ligation efficiency of Gibson assembly with longer DNA and the lower electroporation efficiency of larger plasmids. It is worth noting that the amount of DNA available from the *in vitro* Cas9 digestion of bacterial chromosomes was significantly less than what would be available for typical Gibson assembly: ∼1.5 × 10^7^ cells are embedded in an agarose gel plug, resulting in only a few nanograms of DNA of the desired length. Furthermore, the efficiency of CATCH cloning can be affected by other factors related to the cleavage efficiency of Cas9, for example, the design of the sgRNA sequences^27^. Recent studies showed that the use of truncated or extended versions of guide RNA can reduce the off-target effects of Cas9 nucleases^28^,^29^. In the current study, we have used a variety of PAM sequences (Supplementary Table 3), and the cleavage and cloning results suggested that the selection of PAM sequences (including their orientations with respect to the target genome segment) did not appear to affect the in-gel cleavage and cloning efficiency of CATCH (Fig. 4a, Table 1 and Supplementary Table 2). The off-target effect of Cas9 is less concerning in the cloning of large gene clusters from bacteria genomes; however, this could be a concern when CATCH is applied to other complex genome systems in the future.

To understand the mechanisms responsible for the generation of negative clones, we analysed the plasmids purified from the negative clones by Sanger sequencing, which suggested that most negative clones arose from self-ligation of vectors at sites that share identical sequence of ≥6 bp (Supplementary Fig. 3). We reasoned that vectors nicked at the sites with identical sequences were chewed by the T5 5′–3′ exonuclease, annealed and self-ligated in the Gibson assembly mix; these small, self-ligated plasmids, containing the origin of replication and antibiotic resistance gene but without the target DNA insert, have higher electrotransformation efficiency and faster replication rate than those with long inserts. Thus, we avoided long identical sequences (≥6 bp) on the vector when designing target sites and purified the vectors from PCR products with gentle handling (by pipetting slowly with wide-bore tips).

The ability of CATCH to isolate near-arbitrary DNA segments from the known, flanking sequences may greatly accelerate efforts to fill the gaps in sequenced genomes. Adaptations of CATCH to larger genomes would make it a powerful tool, enabling numerous applications such as targeted cloning of large genes from plants and mammals, targeted sequencing of disease-specific genes in humans or identification of copy-number variations. Efforts to quantify and optimize the specificity and efficiency, and reduce the off-target effects of the CRISPR-Cas9 system^30^,^31^,^32^, as well as discoveries of other targeted nuclease systems with improved specificity should be made to facilitate the application of CATCH to other areas.

While this paper was being reviewed, an alternative method for cloning long genomic DNA segments on the basis of in-solution Cas9 digestion of purified human genomic DNA and yeast TAR was published^33^. While the in-solution digestion by Cas9 is more convenient to carry out, the CATCH in-gel cleavage protects chromosomal DNA from shearing, permitting the cloning of longer DNA sequences of up to 150 kb. Furthermore, another apparent advantage of using the in-gel Cas9 cleavage instead of in-solution digestion of purified genomic DNA is that, because the rest of the genomic DNA is protected by the agarose gel matrix, the background DNA fragments that could be falsely ligated and cloned, leading the generation of negative clones, can be greatly reduced. In addition, using in-gel cleavage as opposed to in-solution digestion allows for immediate PFGE assessment of the cleavage efficiency (Supplementary Fig. 1), and thus makes the troubleshooting and optimization of the Cas9 cleavage feasible. Although TAR uses long hooks of up to a few hundred bp for homologous recombination, the cloning has to be carried out *in vivo* in yeast, and an additional step would be required to clone the inserts into BAC for maintenance and further genetic manipulation. The use of *in vitro* Gibson assembly in CATCH, on the other hand, permits one-step ligation and cloning into BAC to be accomplished. Future adaptations of both methods, for example, combining the in-gel digestion method of CATCH and TAR, may facilitate efforts to clone large gene clusters from bacteria, as well as long genome segments from other organisms.

## Methods

### Strains and plasmids

*E. coli* str. K-12 substr. MG1655 was obtained from the laboratory of Professor Guoqiang Chen (Tsinghua University). *E. coli DH5a* str. WPN25-sfGFP was obtained by integrating an *sfGFP* expression cassette into the corresponding chromosomal integration site (2,875,434–2,875,653) using the One-Step Integration Plasmid^34^. The sequence of the integration site was verified using PCR and sequence analysis. *B. subtilis* str. 168 was obtained from the laboratory of Professor Tingyi Wen (Institute of Microbiology, Chinese Academy of Sciences). *S. venezuelae* ISP52030 and *S. aureofaciens* ATCC 10762 were obtained from the laboratory of Professor Keqian Yang (Institute of Microbiology, Chinese Academy of Sciences). Electrocompetent *E. coli* cells TransforMax EPI300 and BAC vector pCC1BAC were purchased from Epicentre. The p15A vector was provided by Professor Keqian Yang (Institute of Microbiology, Chinese Academy of Sciences).

### Expression and purification of Cas9 and T7 RNA polymerase

The plasmid for *S. pyogenes* Cas9 protein expression was provided by Professor Zhen Xie (Tsinghua University). The histidine-tagged fusion protein Cas9 was purified using a Ni-NTA column and the AKTA system with a Mono S column^18^. In total, ∼10 mg of Cas9 protein was purified and stored in 20 mM HEPES, 150 mM KCl, 1 mM dithiothreitol (DTT), 50% glycerol, pH 7.5 at −20 °C. The plasmid for T7 RNA polymerase expression was provided by Professor Nieng Yan (Tsinghua University) and the enzyme was purified using a Ni-NTA column and the AKTA system with a Mono Q column^35^.

### sgRNA design and preparation

To design the sgRNAs used for in-gel digestion, we first searched for PAM sequence ‘NGG' near the target genomic region, with ‘G' being the starting nucleotide of a transcript because it is ideal for both Cas9 loading^36^ and *in vitro* transcription (IVT) by T7 RNA polymerase. The sgRNA IVT templates were prepared by overlapping PCR of three primers: a primer (X-sgRNA-P) containing the T7 promoter and target sequence, and two others (sgRNA-F and sgRNA-R) containing crRNA–tracrRNA chimera sequence of the sgRNA. All the primers used in this study are listed in Supplementary Table 1. The PCR product was purified by phenol (pH>7.8)/chloroform extraction and isopropanol precipitation, followed by resuspension in RNase-free water. The IVT was performed at 37 °C for 2 h in 100 mM Tris-HCl, pH 8.0, 10 mM MgCl_2_, 30 mM DTT, 2 mM Spermidine, 2.5 mM (each) nucleoside triphosphate (NTP), 10% dimethylsulphoxide, 100 ng μl^−1^ PCR product and 50 μg ml^−1^ T7 RNA polymerase. After incubation, the IVT product was purified by phenol (pH<5.2)/chloroform extraction and isopropanol precipitation. Finally, the sgRNA was resuspended in RNase-free water at a concentration of 300 ng μl^−1^ and stored at −80 °C.

### Genome digestion and recovery

*E. coli* or *B. subtilis* cells were embedded in agarose gel plugs at a concentration of 5 × 10^8^ cells ml^−1^. These plugs were treated with lysozyme, proteinase K and washed with buffer successively according to instructions of the CHEF Bacterial Genomic DNA Plug Kit (Bio-Rad). In the second wash, 1 mM of phenylmethyl sulphonyl fluoride was added to inactivate the residual proteinase K and 0.1 × wash buffer was used for the last wash. The *Streptomyces* mycelia were harvested and used for making gel plugs, as described in ref. 37. The well-washed plugs can be stored in 1 × wash buffer at 4 °C for 2 months, and another round of wash should be performed using 0.1 × wash buffer immediately before Cas9 digestion. For the cleavage reaction, two plugs were first equilibrated at room temperature for 30 min in 1 ml RNase-free cleavage buffer containing 20 mM HEPES, 150 mM KCl, 10 mM MgCl_2_, 0.5 mM DTT and 0.1 mM EDTA at pH 7.5, and then transferred into a new batch of cleavage buffer that contains Cas9 protein (0.1 mg ml^−1^) and the corresponding sgRNA pair (each at 30 ng μl^−1^), and incubated at 37 °C for 2 h. After the reaction, the plugs were washed with 0.1 × wash buffer, and 1/3 of a gel plug was cut out and used to assess the cleavage efficiency by PFGE. The PFGE was performed with 1% agarose gel in 0.5 × TBE using the CHEF Mapper XA System (Bio-Rad) set to auto-algorithm programme with 5–250 kb parameters (6 V cm^−1^, 0.22–21.79 s, 15 h 16 min, 120°) and with circulation at 14 °C. After PFGE, the gel was stained with SYBR Gold (Life Technologies) and the DNA bands were visualized using a ChemiDoc XRS+ Imaging System (Bio-Rad). The rest of the plugs were melted and digested by agarase according to instructions of the GELase Agarose Gel-Digesting Preparation Kit (Epicentre). The digested DNA was precipitated by ethanol and resuspended gently in 20 μl DNase-free water with wide-bore tips. The obtained DNA can be stored at 4 °C for several days, although immediate ligation is recommended.

### Vector ligation and cloning

The vectors were prepared by PCR amplification using pCC1BAC (Epicentre) or p15A vectors as templates, followed by DpnI (NEB) treatment^38^. Each PCR primer consisted of an ∼20-nucleotide (nt) sequence that annealed to the vector template and an ∼30-nt overhang that overlapped with one end of the target DNA. The Gibson assembly mix was prepared as described in the one-step isothermal DNA assembly protocol^9^. A volume of 1 μl vector (∼50 ng) and 4 μl of previously prepared target DNA (with a viscous appearance) were added to 15 μl of Gibson assembly mix by gentle pipetting and incubated at 50 °C for 1 h. After ligation, 2 μl of the mix was transformed into 50 μl TransforMax EPI300 (Epicentre) electrocompetent *E. coli* cells in a 1-mm cuvette (BTX) at 1,300 V using the ECM 399 Electroporation system (BTX). The cells were recovered at 37 °C for 2 h in 1 ml LB medium without antibiotics and then plated on LB medium containing 12.5 μg ml^−1^ chloramphenicol, IPTG and X-gal. After incubation at 37 °C for 12 h, the blue colonies were selected for PCR and sequencing at one of the two junction sites opposite to the *lacZ* gene (with one primer on pCC1BAC (BAC-vF) and the other on the insert (*lacZ*-X kb-vR)). The positive clones were grown in 5 ml LB medium overnight at 37 °C containing 12.5 μg ml^−1^ chloramphenicol and 30 μl CopyControl BAC Autoinduction Solution (Epicentre). Plasmids were extracted from these cells using the QIAprep Miniprep Kit using the QIAcube system (Qiagen) according to the manufacturer's instructions. The purified plasmids were linearized with λ-terminase (Epicentre) and analysed using PFGE as described above. For cloning the *sfGFP*-containing genome segments from *E. coli*, no IPTG or X-gal was added to the LB medium, and the green colonies were selected for PCR and sequencing at one of the two junction sites opposite to the *sfGFP* gene (with one primer on pCC1BAC (BAC-vF) and the other on the insert (*sfGFP*-X kb-vR)). For cloning the *pks* gene cluster from *B. subtilis*, all colonies were PCR- and sequence-verified at both junction sites (Supplementary Fig. 2). The *jad* gene cluster from *S. venezuelae* and *ctc* gene cluster from *S. aureofaciens* were cloned into p15A vector using a similar method, except that the colonies were grown on LB medium with 50 μg ml^−1^ ampicillin and the plasmids were linearized by XbaI.

## Additional information

**How to cite this article:** Jiang, W. *et al*. Cas9-Assisted Targeting of CHromosome segments CATCH enables one-step targeted cloning of large gene clusters. *Nat. Commun.* 6:8101 doi: 10.1038/ncomms9101 (2015).

## Supplementary Material

Supplementary Information Supplementary Figures 1-3 and Supplementary Tables 1-2

## Figures and Tables

**Table 1:** Cloning efficiency of different target sequences and vectors.

Target	Vector	Number of positive colonies	Number of total colonies	Average positive rate±s.d. (%)
*E. coli-lacZ* 50 kb	pCC1BAC	28±4	42±8	65.8±4.0
*E. coli-lacZ* 50 kb (R)	pCC1BAC	26±8	41±7	62.2±9.6
*E. coli-lacZ* 75 kb	pCC1BAC	8±3	23±8	35.1±1.6
*E. coli-lacZ* 100 kb	pCC1BAC	5±3	28±15	21.5±11.2
*E. coli-lacZ* 150 kb	pCC1BAC	1*	51*	1.9*
*E. coli-sfGFP* 50 kb	pCC1BAC	25±6	47±12	55.2±12.4
*E. coli-sfGFP* 75 kb	pCC1BAC	12±4	29±5	39.2±6.4
*E. coli-sfGFP* 100 kb	pCC1BAC	7±3	35±12	21.6±7.4
*B. subtilis-pks* 78 kb	pCC1BAC	4±1	34±9	12.2±3.7
*S. venezuelae-jad* 36 kb	p15A	60±4	66±10	90.5±6.1
*S. aureofaciens-ctc* 32 kb	p15A	70±3	78±6	90.8±3.7

All experiments were performed in triplicates.

^*^Only one positive colony was obtained in three independent trials.
