# Supplementary material for: Cas9-Assisted Targeting of CHromosome segments CATCH enables one-step targeted cloning of large gene clusters
Source: Nat Commun. 2015 Sep 1;6:8101. doi: 10.1038/ncomms9101 (PMC4569707; doi:10.1038/ncomms9101)
Supplement: Supplementary Information — Supplementary Figures 1-3 and Supplementary Tables 1-2 [file ncomms9101-s1.pdf]

Supplementary Figures

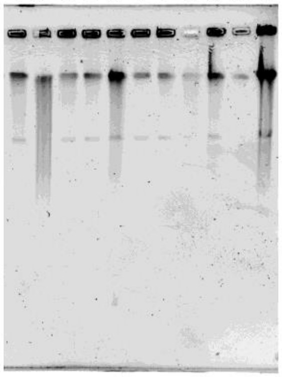

Lane	1	2	3	4	5	6	7	8	9	10	11
Cell concentration (ml^{-1})	5×10^8	5×10^8	5×10^8	5×10^8	5×10^8	5×10^8	5×10^8	5×10^8	5×10^8	5×10^7	5×10^9
Plug volume (μl)	30	30	30	30	30	30	30	15	45	30	30
Incubation time (h)	2	2	2	2	2	1	4	2	2	2	2
Cas9 concentration (mg/ml)	0.1	0.1	0.1	0.02	0.2	0.1	0.1	0.1	0.1	0.1	0.1
sgRNA concentration (ng/μl each)	30	10	100	30	30	30	30	30	30	30	30

Supplementary Figure 1 | Optimization of RNA-guided Cas9 cleavage reaction under different cell concentration, plug volume, incubation time, Cas9 concentration, and sgRNA concentration conditions. Gel plugs containing *E. coli* chromosomes were digested by a pair of sgRNAs targeting a 50 kb genome segment (*E. coli*-*lacZ* 50 kb) under the indicated conditions (shown on the right) and analyzed by PFGE to assess the cleavage efficiency.

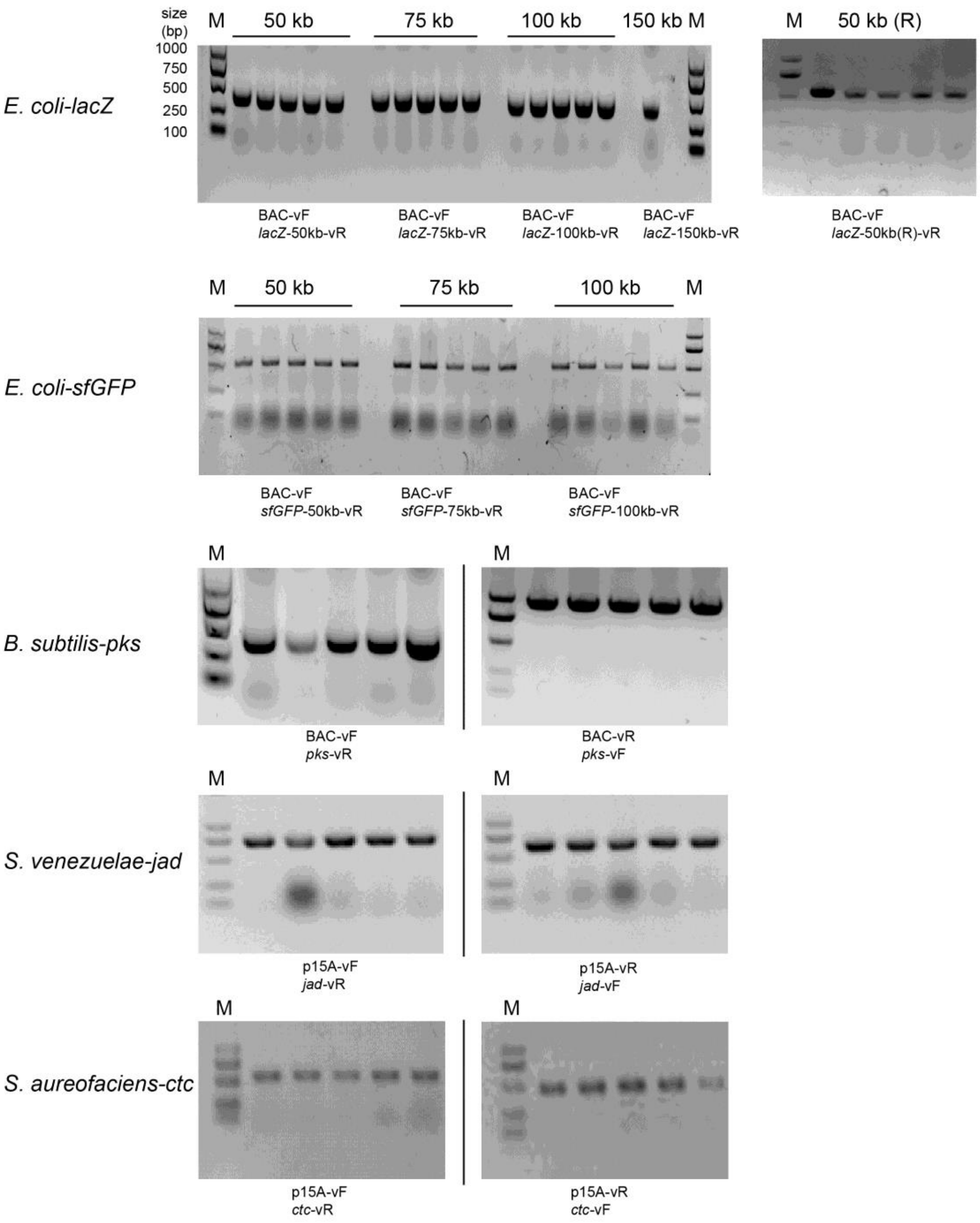

Supplementary Figure 2 | PCR validation of positive clones carrying target inserts cloned from *E. coli*, *B. subtilis*, *S. venezuelae*, and *S. aureofaciens*, respectively. For the blue or the green colonies with target inserts cloned from *E. coli*, PCR was performed at one of the two junction sites opposite to the *lacZ* or *sfGFP* gene, whereas for those from the others, both junction sites were PCR-validated. Names of the PCR primers used are listed below the panels. The same DNA marker was used in all panels. M, marker.

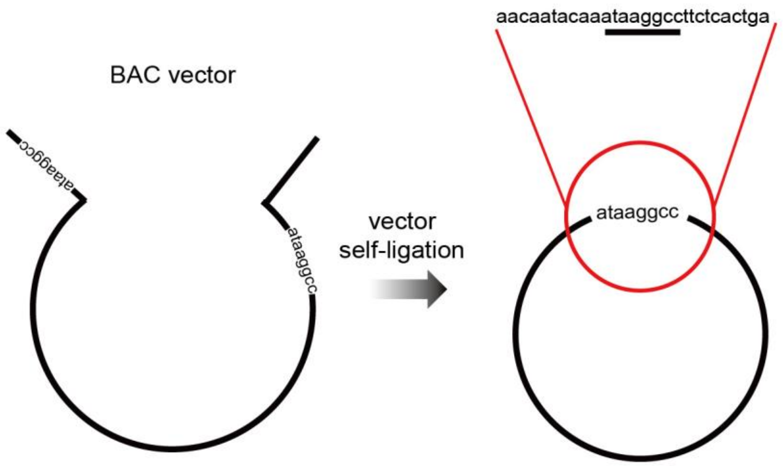

Supplementary Figure 3 | An example of negative clones arising from vector self-ligation at sites with an identical sequence (left: prepared BAC vector with sites that shared 8 bp identical sequence; right: sequencing result of the self-ligation site).

Supplementary Tables

Supplementary Table 1. PCR primers used in this study.

sgRNA template primers	
sgRNA-F	5′-GTTTTAGAGCTAGAAATAGCAAGTTAAAATAAGGCTAGTC-3′
sgRNA-R	5′-AAAAGCACCGACTCGGTGCCACTTTTTCAAGTTGATAACGGACTAGCCTTATTTTAACT-3′
lacZ-sgR1-P	5′-TAATACGACTCACTATAggtgcggatatctcggtagtGTTTTAGAGCTAGAAATAGCAA-3′
lacZ-sgR2-P	5′-TAATACGACTCACTATAggtaggatcataaagtcctcGTTTTAGAGCTAGAAATAGCAA-3′
lacZ-sgR3-P	5′-TAATACGACTCACTATAgaatctgtcgccgaagtaaaGTTTTAGAGCTAGAAATAGCAA-3′
lacZ-sgR4-P	5′-TAATACGACTCACTATAgctgtcggggtgaatttgctGTTTTAGAGCTAGAAATAGCAA-3′
lacZ-sgR5-P	5′-TAATACGACTCACTATAgctgtttacctataatagtcGTTTTAGAGCTAGAAATAGCAA-3′
lacZ-sgR6-P	5′-TAATACGACTCACTATAgtaaatctggggatggcgctGTTTTAGAGCTAGAAATAGCAA-3′
lacZ-sgR7-P	5′-TAATACGACTCACTATAggagaggcggtttgcgtatt GTTTTAGAGCTAGAAATAGCAA-3′
lacZ-sgR8-P	5′-TAATACGACTCACTATAgtgagaaatcgatttttgatGTTTTAGAGCTAGAAATAGCAA-3′
sfGFP-sgR1-P	5′-TAATACGACTCACTATAgtaagttgtatctggagaaaGTTTTAGAGCTAGAAATAGCAA-3′
sfGFP-sgR2-P	5′-TAATACGACTCACTATAgtctcgccaaactggaaaaaGTTTTAGAGCTAGAAATAGCAA-3′
sfGFP-sgR3-P	5′-TAATACGACTCACTATAgccattccgcctgcgctcgaGTTTTAGAGCTAGAAATAGCAA-3′
sfGFP-sgR4-P	5′-TAATACGACTCACTATAgcggacatcgtccagcttgaGTTTTAGAGCTAGAAATAGCAA-3′
sfGFP-sgR5-P	5′-TAATACGACTCACTATAggaactatcaactttttgctGTTTTAGAGCTAGAAATAGCAA-3′
sfGFP-sgR6-P	5′-TAATACGACTCACTATAgcaatcctgttgtgtctgacGTTTTAGAGCTAGAAATAGCAA-3′
pks-sgR1-P	5′-TAATACGACTCACTATAgtaaacagctgcaatcccatGTTTTAGAGCTAGAAATAGCAA-3′
pks-sgR2-P	5′-TAATACGACTCACTATAgcctatgagattcctttattGTTTTAGAGCTAGAAATAGCAA-3′
jad-sgR1-P	5′-TAATACGACTCACTATAggcgaagtccttgcccatgatgGTTTTAGAGCTAGAAATAGCAA-3′
jad-sgR2-P	5′-TAATACGACTCACTATAggacgacgaggatccgacctgaGTTTTAGAGCTAGAAATAGCAA-3′
ctc-sgR1-P	5′-TAATACGACTCACTATAggacccaccggaggacttcgcaGTTTTAGAGCTAGAAATAGCAA-3′
ctc-sgR2-P	5′-TAATACGACTCACTATAggtctccaccgtctaccgcgacGTTTTAGAGCTAGAAATAGCAA-3′
Vector amplification primers	
lacZ-BAC-F	5′-tgagctgtcttcggtatcgtcgtatcccactttattatcacTTATTCAGGCGTAGCAAC-3′
lacZ-50kb(R)-BAC-F	5′-cggccaacgcgcggggagaggcggtttgcgtttattatcacTTATTCAGGCGTAGCAAC-3′

lacZ-50kb-BAC-R	5′-gacatgccaaaagagtggacaacgacccgagGCGGCCGCATCGAATATAA-3′
lacZ-50kb(R)-BAC-R	5′-ttctttatctgtcagtgagaaatcgatttttGCGGCCGCATCGAATATAA-3′
lacZ-75kb-BAC-R	5′-gccgtttcaaatctaacactcgtaatttacccttt GCGGCCGCATCGAATATAA-3′
lacZ-100kb-BAC-R	5′-agcaacgactgatagtagtatcttccccagcGCGGCCGCATCGAATATAA-3′
lacZ-150kb-BAC-R	5′-ttttgctgccaccagatttgcgccgcccgacGCGGCCGCATCGAATATAA-3′
lacZ-200kb-BAC-R	5′-ccaggtctccgtaaccgcaggcagccccagcGCGGCCGCATCGAATATAA-3′
sfGFP-BAC-F	5′-ctgttaaaaaacgattataaaatttcccttttattatcacTTATTCAGGCGTAGCAAC-3′
sfGFP-50kb-BAC-R	5′-tatgtggattaatcagatgtgtttgccatttGCGGCCGCATCGAATATAA-3′
sfGFP-75kb-BAC-R	5′-cggcgcagaaccgagtacatcacggccatcgGCGGCCGCATCGAATATAA-3′
sfGFP-100kb-BAC-R	5′-aaaaagattgatgcgattataaaacccttcaGCGGCCGCATCGAATATAA-3′
sfGFP-150kb-BAC-R	5′-aacgtcgaagatggtcgtttcgttgcccagcGCGGCCGCATCGAATATAA-3′
sfGFP-200kb-BAC-R	5′-gggcaatgaaaaaatcgccagggtacctgtcGCGGCCGCATCGAATATAA-3′
pks-BAC-F	5′-gttttcttggtgaatatgaagctcacctaatttattatcacTTATTCAGGCGTAGCAAC-3′
pks-BAC-R	5′-taccgcggagcctcagcgaccgcagcccatgGCGGCCGCATCGAATATAA-3′
jad-p15A-F	5′- agtgccacaagcgtctaggggagctccacatGGTGAAGATCCTTTTTGATAATCTCATG -3′
jad-p15A-R	5′- cggcggaggtgccgtggaagccgggccgtcaTAGATCCTTTTGGTTCATGTGCAGCTC -3′
ctc-p15A-F	5′- gcctctggccggccggggaaagcagccatgcGGTGAAGATCCTTTTTGATAATCTCATG 3′
ctc-p15A-R	5′- gcaggtgggtgagggtgtcggtcatcccgtcTAGATCCTTTTGGTTCATGTGCAGCTC-3′
Validation primers	
BAC-vF	5′-agtccgagctcatcgctaat-3′
BAC-vR	5′-ggatagtgttcacccttgttaca-3′
lacZ-50kb-vR	5′-gcattttgattcacagcagtca-3′
lacZ-50kb(R)-vR	5′-ggtgacgctcatcatggtt-3′
lacZ-75kb-vR	5′-gacgataaccttagagggatgat-3′
lacZ-100kb-vR	5′-cgagctttaatgcctctgct-3′
lacZ-150kb-vR	5′-attcctgtgccttaatgacaat-3′
sfGFP-50kb-vR	5′-gcattctgtttcacacgacga-3′
sfGFP-75kb-vR	5′-gcagggtctgtttacgcca-3′
sfGFP-100kb-vR	5′-ttgtccgcgcgttctcta-3′
pks-vF	5′-ccatacaatcatcgtatcgggt-3′

pks-vR	5′-ccctccatccctcgttctaa-3′
p15A-vF	5′-gagtccaacccggtaagacacgac-3′
p15A-vR	5′- gagcgtccctcccggacc-3′
jad-vF	5′-acggacgagatccacacgg-3′
jad-vR	5′-tcgccctggccctggacag-3′
ctc-vF	5′- gaccgagagcgcggccacc-3′
ctc-vR	5′- tggcgacaggcgcgagtga-3′

Supplementary Table 2. Quantitative results and positive rates of the performed CATCH cloning experiments.

Target	Vector	Trial	# of positive colonies	# of total colonies	Positive rate	Average positive rate ± s.d.
E. coli-lacZ 50 kb	pCC1BAC	1	31	47	66.0%	65.8±4.0%
		2	23	33	69.7%	
		3	29	47	61.7%	
E. coli-lacZ 50 kb (R)	pCC1BAC	1	25	42	59.5%	62.2±9.6%
		2	19	35	54.2%	
		3	35	48	72.9%	
E. coli-lacZ 75 kb	pCC1BAC	1	8	22	36.4%	35.1±1.6%
		2	11	31	35.5%	
		3	5	15	33.3%	
E. coli-lacZ 100 kb	pCC1BAC	1	8	24	33.3%	21.5±11.2%
		2	3	15	20.0%	
		3	8	35	22.9%	
E. coli-lacZ 150 kb	pCC1BAC	1	0	30	0	N/A
		2	0	23	0	
		3	1	51	1.9%	
E. coli-lacZ 200 kb	pCC1BAC	1	0	10	0	N/A
		2	0	23	0	
		3	0	17	0	
E. coli-sfGFP 50 kb	pCC1BAC	1	24	58	41.3%	55.2±12.4%
		2	32	49	65.3%	
		3	20	34	58.8%	
E. coli-sfGFP 75 kb	pCC1BAC	1	12	29	41.3%	39.2±6.4%
		2	8	25	32.0%	
		3	15	34	44.1%	
E. coli-sfGFP 100 kb	pCC1BAC	1	5	24	20.8%	21.6±7.4%
		2	7	48	14.6%	
		3	10	34	29.4%	
E. coli-sfGFP 150 kb	pCC1BAC	1	0	23	0	N/A
		2	0	38	0	
		3	0	28	0	
E. coli-sfGFP 200 kb	pCC1BAC	1	0	25	0	N/A
		2	0	36	0	
		3	0	48	0	
B. subtilis-pks 78 kb	pCC1BAC	1	4	25	16.0%	12.2±3.7%
		2	3	35	8.6%	
		3	5	42	11.9%	
S. venezuelae-jad 36 kb	p15A	1	55	57	96.5%	90.5±6.1%
		2	60	66	90.9%	
		3	64	76	84.2%	
S. aureofaciens-ctc 32	p15A	1	68	72	94.4%	90.8±3.7%

kb		2	70	77	90.9%	
		3	74	85	87.1%	

Supplementary Table 3. Target sequences used in CATCH cloning, including the PAM motifs.

sgRNA	Target sequence + PAM sequence (capital characters: NGG)	Location of PAM
lacZ-sgR1	ggtgcggatatctcggtagtGGG	target genome segment
lacZ-sgR2	ggtaggatcataaagtcctcGGG	target genome segment
lacZ-sgR3	gaatctgtcgccgaagtaaaGGG	target genome segment
lacZ-sgR4	gctgtcggggtgaatttgctGGG	target genome segment
lacZ-sgR5	gctgtttacctataatagtcGGG	target genome segment
lacZ-sgR6	gtaaatctggggatggcgctGGG	target genome segment
lacZ-sgR7	ggagaggcggtttgcgtattGGG	flanking sequence
lacZ-sgR8	gctgaccgggctgaacttccGGG	flanking sequence
sfGFP-sgR1	gtaagttgtatctggagaaaGGG	target genome segment
sfGFP-sgR2	gtctcgccaaactggaaaaaTGG	target genome segment
sfGFP-sgR3	gccattccgcctgcgctcgaTGG	target genome segment
sfGFP-sgR4	gcggacatcgtccagcttgaAGG	target genome segment
sfGFP-sgR5	ggaactatcaactttttgctGGG	target genome segment
sfGFP-sgR6	gcaatcctgttgtgtctgacAGG	target genome segment
pks-sgR1	gtaaacagctgcaatcccatGGG	target genome segment
pks-sgR2	gcctatgagattcctttattGGG	target genome segment
jad-sgR1	cgaagtccttgcccatgatgTGG	target genome segment
jad-sgR2	acgacgaggatccgacctgaCGG	target genome segment
ctc-sgR1	acccaccggaggacttcgcaTGG	target genome segment
ctc-sgR2	tctccaccgtctaccgcgacGGG	target genome segment
